# The Potential of Metabolomic Analyses as Predictive Biomarkers of Preterm Delivery: A Systematic Review

**DOI:** 10.3389/fendo.2021.668417

**Published:** 2021-09-06

**Authors:** Emma Ronde, Irwin K. M. Reiss, Thomas Hankemeier, Tim G. De Meij, Nina Frerichs, Sam Schoenmakers

**Affiliations:** ^1^Division of Obstetrics and Prenatal Diagnosis, Erasmus University Medical Centre, Rotterdam, Netherlands; ^2^Department of Pediatrics, Division of Neonatology, Erasmus University Medical Centre, Rotterdam, Netherlands; ^3^Division of Analytical Biosciences, Leiden Academic Centre for Drug Research, Leiden University, Leiden, Netherlands; ^4^Department of Pediatric Gastroenterology, Amsterdam University Medical Centre, Amsterdam, Netherlands

**Keywords:** preterm delivery, metabolomics, microbiota, VOC, metabolites, biomarkers

## Abstract

**Scope:**

as the leading cause of perinatal mortality and morbidity worldwide, the impact of premature delivery is undisputable. Thus far, non-invasive, cost-efficient and accurate biochemical markers to predict preterm delivery are scarce. The aim of this systematic review is to investigate the potential of non-invasive metabolomic biomarkers for the prediction of preterm delivery.

**Methods and Results:**

Databases were systematically searched from March 2019 up to May 2020 resulting in 4062 articles, of which 45 were retrieved for full-text assessment. The resulting metabolites used for further analyses, such as ferritin, prostaglandin and different vitamins were obtained from different human anatomical compartments or sources (vaginal fluid, serum, urine and umbilical cord) and compared between groups of women with preterm and term delivery. None of the reported metabolites showed uniform results, however, a combination of metabolomics biomarkers may have potential to predict preterm delivery and need to be evaluated in future studies.

## Introduction

Preterm delivery is defined as any delivery occurring before 37 + 0 weeks of gestation, of which around two-thirds is considered to be spontaneous (such as after spontaneous rupture of membranes) and one-third is considered iatrogenic (for example emergency caesarean section for suspicion of fetal distress) (1). Spontaneous preterm delivery in (a)symptomatic pregnant women has a multifactorial origin and the largely unknown aetiology and pathophysiology makes it challenging to predict who is actually at risk to deliver preterm.

**Figure 1:**
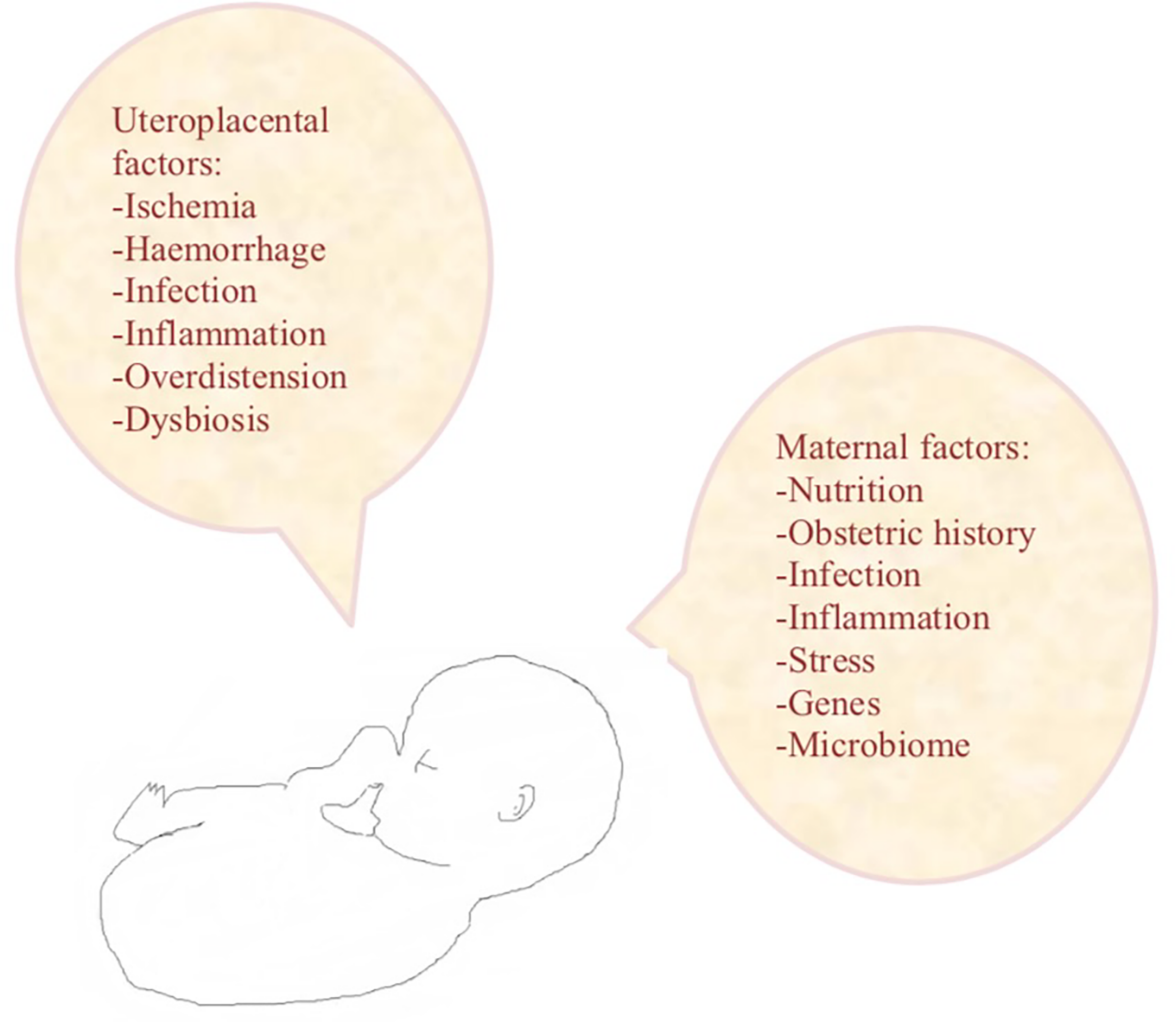
Risk factors for preterm labour.

In the industrialised world, the incidence of preterm labour continues to rise despite increasing knowledge on risk factors (1). Maternal risk factors include nutritional status, obstetric history, characteristics of the ongoing pregnancy, psychological factors, genetic and biological predisposition, stress, infection and inflammation. Uteroplacental risk factors include ischaemia or haemorrhage, uterine overdistension and decreased cervical length (**Figure 1**). Over the past years, increasing evidence exists that the disturbances of the commensal microbiota and consequently alterations in immunologically mediated processes may provoke preterm labour (1). In contrast to the aged hypothesis of the sterile, and as such presumed healthy, anatomical environments, the uterine cavity and urine of healthy pregnant woman harbour a unique, symbiotic microbiome (1). Disturbances in the healthy symbiotic composition can lead to an unhealthy balance, a dysbiosis, possibly initiating an inflammatory cascade inducing preterm birth (1).

The lack of predictive biomarkers hampers the implementation of timely and effective intervention options to prevent preterm delivery. A dysbiotic state can potentially be detected or evaluated using targeted biomarkers. Preterm delivery biomarker discovery has progressed over the years as novel analytical techniques, including proteomics, namely nuclear magnetic resonance (NMR) spectroscopy and mass spectrometry (MS) have emerged. Also, more portable diagnostic devices have emerged such as electronic-nose (e-nose) instruments, which are gas-sensing systems that may detect disease-associated volatile organic compounds (VOCs), reflecting microbiota composition and function (2). An alteration of a local metabolic state, such as caused by infection or a change in microbiome composition, will lead to a change in emitted gaseous chemicals, VOCs. Metabolomics is the study of an altered metabolic state of substrates and products of their chemical reactions, the socalled metabolites. Metabolites are involved in signalling pathways such as the stimulation and inhibition of enzymes, defence and growth, development and reproduction. If metabolites, found in both the physiologic (term delivery) and pathologic (preterm delivery) conditions, can correctly be isolated, identified and interpreted, they will provide insight into the metabolic pathways resulting in de pathophysiological cascade causing preterm birth. Systemic or local inflammation processes and changes in microbiota equilibrium leading to preterm labour most likely produce VOCs that are not present in healthy pregnant individuals. Benefits of the long term clinical application of metabolomics will include patient friendly non-invasive collection and real time-analyses (such as urine or fecal samples), possible resulting in shorter and as such less-expensive, stays in hospital care and earlier more effective treatments. Potential short term disadvantages include complexity in interpretation by lack of standardization and time-consuming metabolic profiling.

Every year, an estimated 15 million babies are born preterm worldwide making this the leading cause of child mortality under five years of age (3). With preterm birth being the leading cause of perinatal mortality and morbidity, the health care system would benefit from identification of reliable predictors, with significant clinical and health care expenditure impact. In a recent systematic review (4) societal economic burden associated with pre-maturity (≤36 weeks of gestation) in the US was at least 26.2 billion USD annually in 2005, or 51,600 USD per infant, and an inverse relationship between hospital costs, neonatal birth weight and gestational age has been defined (5). From a maternal perspective, in an American cohort of 2534 pregnant women, 9% had hospital admissions for imminent preterm labour of which only 38% actually gave birth during their first admission (6). The remaining 72% of the women who initially were diagnosed with preterm labor and did not deliver immediately were admitted to receive antenatal corticosteroids and tocolytics. Despite years of research, reliable prediction methods for preterm labour are scarce and yet urgently needed to reduce costs, mortality and morbidity.

The goal of the current systematic review is to present an overview of described metabolites associated with preterm birth in (a)symptomatic pregnant women as potential biomarkers for the adequate and timely prediction of preterm birth. Based upon national and international guidelines (NVOG, RCOG), we focused on recommended samples collected during a clinical work up for (imminent) preterm birth, which involves maternal samples of blood, urine, vaginal and cervical fluid and fetal compartment samples of amniotic fluid and umbilical cord blood.

We aim to answer the following questions: 1.) Can metabolomics be used to predict premature birth in (a)symptomatic pregnant women? 2.) Which metabolites are associated with premature birth in (a)symptomatic pregnant women? 3.) Which sources of samples (amniotic fluid, vaginal fluid, blood, urine) are most useful in predicting premature birth in (a)symptomatic pregnant women?

## Methods

### Search Strategy

We searched the following databases: Medline, Embase, The Cochrane Library, Cochrane Central Register of Controlled Trials, Cochrane Methodology Register, PubMed, Google Scholar and Web of Science. The search strategy is available as a supplement (**Supplementary Material 1**). The protocol for this systematic review was designed and registered *a priori* at the PROSPERO registry (CRD42020155742).

### Systematic Review Protocol

Results were restricted to human studies alone and included only articles in the English language. Databases were searched from inception to May 2020. Preterm delivery was defined as a gestational age preceding 37 + 0 weeks of gestation. We excluded letters, conference abstracts, editorials, systematic reviews and meta-analyses. Metabolites were identified, defined and included when present in the online accessible Human Metabolome Database (7). Analysed human samples included for the current review were maternal vaginal/cervical swabs, urine, blood, amniotic fluid and placentas as well as fetal/neonatal urine and umbilical cord blood. The main outcome was the detectable presence of metabolites within these samples under varying medical circumstances associated with preterm delivery, such intra-amniotic infection and premature rupture of the membranes, but also preterm birth itself, in comparison with full term deliveries and women without signs of infection.

### Data Extraction

The search was done by an independent employee of the medical library of the Erasmus MC (WB). Subsequently, two reviewers screened the articles for inclusion in the systematic review. Differences were resolved by discussion, and when reviewers did not reach agreement, the article was referred to a third reviewer. Data extracted included the year of publication, study design, study population, sample size, outcome data, exclusion criteria, potential confounders, results and conclusion.

## Results

### Risk of Bias

A quality assessment was conducted on each of the included studies. This quality score can be used to assess the quality of studies included in systematic reviews and meta-analyses and is applicable for both interventional and observational studies. The score was designed and developed by the Rotterdam Intergenerational Ageing Research Center (www.erasmusage.com) at the Erasmus Medical Centre. The quality score is composed of 5 items, and each item is allocated 0, 1 or 2 points. This allows a total score between 0 and 10 points, 10 representing the highest quality.

### Studies Retrieved

**Figure 2:**
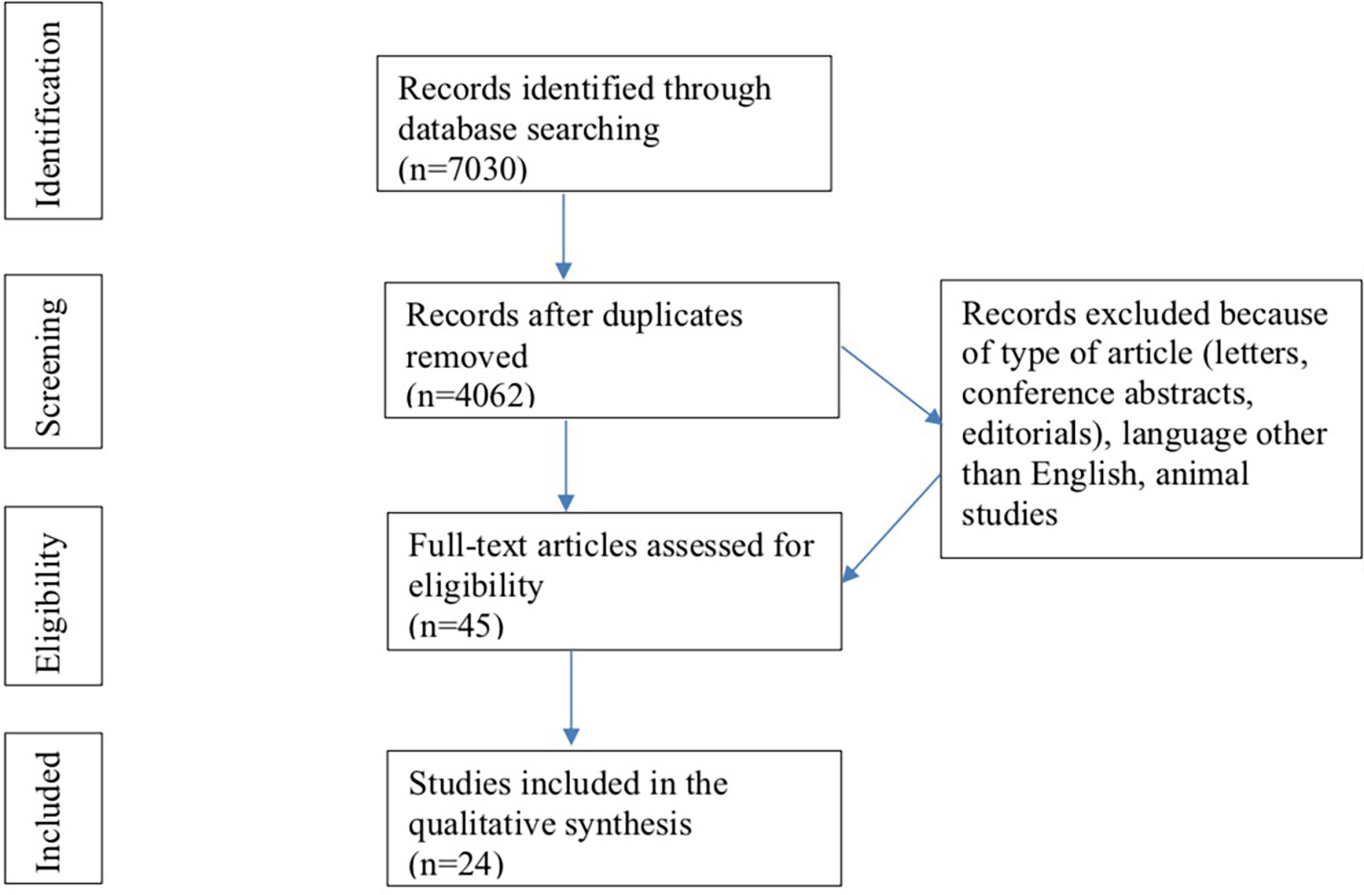
Flowchart of in- and excluded studies.

The flowchart summarizes the process of literature search and selection of studies included (**Figure 2**). The initial search identified 7030 records of which 2968 were duplicates. Of the remaining 4062 records, 4017 were excluded because they involved studies conducted in animals, did not fit the inclusion or search criteria or were not written in the English language. Ultimately, the full text of 45 articles was read, after which 21 papers were excluded based on the predefined in- and exclusion criteria, resulting in 24 articles for analyses. Of these studies 8 are case-control and 16 are cohort studies (both prospective and retrospective). The metabolites included in this systematic review are described in **Table 1** and based on the Human Metabolome Database.

**Table 1:** Included metabolites based on the Human Metabolome Database (7).

Type of metabolite	Mechanism of action	Found in/origin
Acetate	Depending on type of acetate energy source, membrane stabilizer	Food
Adenosine monophosphate-activated kinase	Plays a role in lipid metabolism and glucose transport	Small amounts in the liver
ADP-Mannose	Catalyzes the hydrolysis of a variety of nucleoside diphosphate derivatives	Erythrocytes, placenta and liver
Alkanes (decane, undecane, dodecane)	Potential biomarker for consumption of foods such as herbs. Naturally occurring process: in asthma	Food, endogenous
Cortisol and DHEAS	Involved in stress response	Endogenous (main glucocorticoid secreted by adrenal cortex) and food
D-Galactose	Basic substrate for the biosynthesis of many macromolecules in the body. Found in the urine it is a biomarker for the consumption of milk.	Lactose in the milk of mammals, some fruit and vegetables
Ferritin	Storage molecule to sequester body iron	Food, endogenous such as in the brain and liver
Formate	Biological role: nutrient	Food, subcellular
Fructose	Involved in metabolism (amino sugar, starch and sucrose, galactose)	Food
Glutamate	Incomplete breakdown product of protein digestion and protein catabolism	Endogenous
25-Hydroxyvitamin D2	Membrane stabilizer, energy source, signalling molecule	Food, endogenous
8-Isoprostane	Elevated in asthma exacerbations	Endogenous (skin, adipose tissue, platelet, membrane)
Lactate	Involved in pyruvate metabolism and pyruvaldehyde degradation	Food, tissues
Lysine	Health effect: metabolism and nutrition disorders (aciduria, hyperlysinemia)	Endogenous (nerve cell, cytoplasm, faeces)
NAD(P)H oxidase	Functions as an electron carrier amongst others in the Calvin cycle, cholesterol synthesis, fatty acid elongation, and nucleic acid synthesis	All tissues
Nitrate	Involved in enterohepatic metabolism	Food, endogenous
Nitrite	Signaling molecule, osmolyte	Food, endogenous
4-Phenylbutyric acid (4-PBA)	Treats elevated blood ammonia in urea cycle disorders	Subcellular: membrane, cell membrane. In the pancreas
Phthalate	May exhibit weak estrogenic activity	Food
Progesterone	Decreases contractility of uterine smooth muscle, supports gestation, inhibits lactation during pregnancy, involved in the female menstrual cycle. A drop is one step that facilitates the onset of labour	Secreted primarily by the corpus luteum and the placenta
Prostaglandin E2	Stimulates bone resorption by osteoclasts, induces fever, increases vasodilation and cAMP production, enhances bradykinin and histamine, induces uterine contractions, platelet aggregation, maintains the opening of the fetal ductus arteriosus	Synthesis in all mammalian cells except erythrocytes.
Prostaglandin F2 alpha	Contraction of pulmonary arteries. Modulation of intraocular pressure and smooth muscle contraction in the uterus and gastrointestinal tract sphincters	Synthesis in all mammalian cells except erythrocytes.
Succinate	Involved in lipid metabolism, transport and fatty acid metabolism	Food, endogenous
Tauroursodeoxycholic acid (TUDCA)	Prevents apoptosis and protects mitochondria from cellular elements that would otherwise interfere with energy production	Formed in the liver, is a bile acid
Vitamin A	Important in vision and for bone growth	Food, enriched in the liver, egg yolks and the fat component of dairy products
Vitamin C	Necessary for maintaining connective tissue and bone. Reducing agent in several metabolic pathways	Citrus fruits and many vegetables
Vitamin E	Involved in lipid transport, lipid metabolism, fatty acid metabolism	Food, endogenous

The discussed metabolites are displayed in **Table 2** and are presented according to the sources of samples (amniotic fluid, vaginal fluid, blood, placenta, urine).

**Table 2:** Study selection and characteristics.

Amniotic fluid									
Author	Year	Reference	Source	Metabolite	Effect		ErasmusAGE score	Study type	Study country
Lee	2016	(8)	Amniotic fluid	PGF2 alpha	Elevated concentration in preterm labor, IAI, spontaneous preterm delivery		5	Prospective cohort	South Korea
Musilova	2015	(9)	Amniotic fluid	PGE2	The median concentration was significantly higher in cases with acute chorioamnionitis		5	Prospective cohort	Czech Republic
Park	2016	(10)	Amniotic fluid	PGF alpha	The concentration of was increased in the AF of 40% of patients with preterm labour and intact membranes and is an independent risk factor IAI		6	Retrospective cohort	South Korea
Romero	2012	(11)	Amniotic fluid	Mannose, D-galactose and fructose	Decreased in patients with preterm labour and intra-amniotic infection	6		Retrospective cross-sectional study	USA and Chile
**Placenta**									
Furuta	2000	(26)	Placentas	PGE2	Five inflammatory cytokines stimulated PGE2 production from amnion cells		4	Prospective cohort	Japan
Garcia- Ruiz	2015	(27)	Placentas	Progesterone	Progesterone significantly blunted cytokine secretion of LPS treated placentas		4	Prospective cohort	Mexico
Lim	2015	(28)	Placentas	AMPK	AMPK was decreased in term labor. No effect in preterm labor. The activity was decreased in preterm fetal membranes in the absence of labor, with PROM compared to intact membranes.		4	Prospective cohort	Australia
L Liong	2014	(29)	Placentas	TUDCA	The increase in ER stress by LPS stimulation was reversed using ER stress inhibitors 4-PBA and TUDCA		4	Prospective cohort	Australia
Matsubara	2001	(30)	Placentas	NADPH	In fetal membranes without chorioamnionitis, 25% showed NADPH oxidase activity, in mild CAM 51% showed activity		4	Prospective cohort	Japan
Stuart	2005	(20)	Placentas	Vitamin c	Lower concentration of ascorbic acid in amniotic tissue from PROM women compared with controls		5	Prospective cohort	UK
**Umbilical cord**									
Buhimschi	2008	(31)	Umbilical cord blood	Cortisol	Median concentration not significantly different in fetuses with and without intraamniotic inflammation		4	Prospective cohort	USA
**Vaginocervical fluid**									
Amabebe	2016	(19)	Vagino- cervical fluid	Acetate	Acetate appears to be predictive of preterm delivery, the women who delivered prematurely had a twofold higher acetate level than those who delivered at term		4	Case-control	UK
Dorfeuille	2016	(15)	Vaginal fluid	Lactate	Significant association with the risk of chorioamnionitis		4	Prospective cohort	Canada
Giannella	2011	(14)	Vaginal fluid	Nitric oxide	Strong association between cervical and gingival NO levels and preterm delivery		6	Case-control	Italy
Nakatsuka	2000	(12)	Vaginal fluid	Nitrite and nitrate	The elevation in vaginal secretions is accompanied by premature rupture of membranes and it precedes premature deliver		3	Prospective cohort	Japan
Ramsey	2002	(16)	Cervical fluid	Ferritin	Elevated cervical ferritin levels are associated (in asymptomatic women) with subsequent preterm birth		4	Case-control	USA
Stafford	2017	(18)	Vaginal fluid	Succinate	Women who deliver preterm had significantly lower succinate levels compared to women who delivered at term		6	Case-control	UK
Vaisanen	2003	(13)	Cervical fluid	Nitric oxide	Nitric oxide metabolite level rises after cervical ripening, or cervical manipulation		3	Prospective cohort	Finland
Broumand	2014	(17)	Cervicovaginal fluid	Ferritin	Ferritin levels are higher in preterm deliveries		6	Prospective cohort	Iran
**Blood**									
Akkar	2016	(22)	Blood	Vitamin D	Decreased in preterm group		5	Case-control	Turkey
Bhupornvivat	2017	(23)	Blood	Vitamin D	No difference between preterm (20.9 ng/ml ± 8.4) vs control (21.2ng/ml ± 6.7) (p 0.91)		4	Case-control	Thailand
Ilhan	2015	(21)	Blood	Vitamin C	Low levels in PPROM (7.29mg/L ± 2.19) vs control (13.85mg/L ± 3.07) (p 0.0012)		5	Case-control	Turkey
Ilhan	2015	(21)	Blood	Vitamin E	No effect (20.38mg/L ± 6.47) vs control (18.15mg/L ± 6.38) (p 0.15)		5	Case-control	Turkey
Ilhan	2015	(21)	Blood	Vitamin A	No effect (0.70mg/L ± 0.57) vs control (0.76mg/L= ± 0.44) (p 0.65)		5	Case-control	Turkey
Ilhan	2015	(21)	Blood	8-isoprostane	Lower levels in PPROM (55.6pg/L ± 146.2) vs control (130.2pg/L ± 161.6) (p 0.0011)		5	Case-control	Turkey
**Urine**									
Ferguson	2014	(24)	Urine	Phthalate	Increase		3	Retrospective cohort	Puerto Rico
Maitre	2014	(25)	Urine	Lysine	Elevation in spontaneous preterm delivery		5	Prospective cohort	Greece
Maitre	2014	(25)	Urine	Formate	Decrease in spontaneous preterm delivery		5	Prospective cohort	Greece

### Synthesized Findings

#### Amniotic Fluid

##### Prostaglandins

Four studies looked at prostaglandin as a biomarker for preterm labour in a total of 472 women.

It has previously been suggested that elevated prostaglandin levels are associated with culture-proven intra-amniotic inflammation (IAI). In the first study, it was investigated whether the concentration of the metabolite prostaglandin PGF2 alpha (1) could serve as a prognostic factor for impending delivery and (2) allow for detection of patients with culture-negative amniotic fluid (AF) infection. IAI was defined as a positive AF culture and/or increased concentration of AF matrix metalloproteinase-8 (MMP-8) concentration, a protein involved in the breakdown of extracellular matrix, tissue remodelling and disease processes where earlier studies indicated that it is a sensitive and specific index of inflammation. An IAI with culture-negative AF was defined as a matrix metalloproteinase-8 concentration of >32ng/ml. The concentration of PGF alpha was examined in the amniotic fluid (obtained by transabdominal amniocentesis or caesarean section) of women with preterm premature rupture of the membranes (PPROM) (≤35+0 weeks) in singleton pregnancies (n=140). Subsequently, the AF was cultured and women were divided into three groups depending on AF culture results and whether IAI was present. Group 1 included patients without IAI and a negative AF culture (N=81), group 2 included women with an IAI and negative AF culture (N=31), and group 3 included patients with a positive AF culture (N=28).

Women with a high AF PGF2 alpha concentration (>170 pg/mL) at the moment of collection had a significantly lower median gestational age at delivery, a shorter time to delivery, and a higher frequency of preterm delivery before 36 + 0 weeks of gestation than patients with a lower AF PGF2 alpha (< 170 pg/mL) concentration. The presence of women with IAI and negative AF cultures indicates either a detection problem or a source of extra-uterine infection or non-infection related mechanisms resulting in an elevation of PGF2 alpha concentrations in AF, such as the activation of the hypothalamic pituitary-adrenal (HPA) axis of the fetus. In line with these findings, in a retrospective cohort study, Park et al. (10) included 132 women with singleton pregnancies with a gestational age of 20 + 0 until 35 + 0 weeks and regular uterine contractions (defined as eight or more within 60 minutes with intact membranes). AF was collected by amniocentesis or during caesarean section and was cultured for bacteria and genital mycoplasmas. The presence of IAI was also defined as the detection of an elevated MMP-8 concentration. Finally, histopathologic examination of the placentas was performed; chorioamnionitis was defined as the placental presence of acute inflammatory changes. They reported that 40.2% (53/132) of patients with preterm labour and intact membranes had an elevated PGF alpha concentration, which was associated with IAI in 49% (26/53) versus 20% (16/79) and chorioamnionitis. Patients with an elevated PGF alpha concentration had a significantly shorter amniocentesis-to-delivery interval compared with those with a normal AF PGF alpha concentration (hazard ratio 2.1% with a 95% confidence interval of 1.4-3.1, p=0.001).

A different type of prostaglandin, prostaglandin E2 (PGE2) was studied by Musilova et al. (9) This prospective cohort study included 145 women with singleton pregnancies complicated by PPROM between 24 + 0 and 36 + 6 weeks of gestational age with respect to microbial invasion of the amniotic cavity (MIAC), intraamniotic inflammation (IAI), microbial-associated IAI, histological chorioamnionitis and short-term neonatal morbidity. Amniotic fluid was obtained by amniocentesis. IAI was defined as amniotic fluid interleukin-6 concentrations higher than 745 pg/mL and MIAC was defined as a positive PCR for Ureaplasma, M. hominis and/or for C. trachomatis and/or by positivity for the 16S rRNA gene. The diagnosis of histological chorioamnionitis was based on histopathological examination of amnion and chorion-decidua, placenta and the umbilical cord. This study showed that AF PGE2 concentrations were not different in women with and without MIAC, women with IAI had higher AF PGE2 concentrations than those without IAI and women with HCA had higher AF PGE2 concentrations than those without HCA. The authors hence suggest that elevation of PGE2 is the result of an intraamniotic inflammatory response either to infectious or to non-infectious stimulus, but not MIAC per se.

##### Prostaglandins, Carbohydrates and Amino Acids

In line with Musilova et al. (9), Romero et al. (11) did not observe a difference in prostaglandin levels in this metabolic profiling study of AF, however the type of prostaglandin was not specified. AF was obtained transabdominally through amniocentesis and analysed using a combination of liquid chromatography and gas chromatography coupled with mass spectrometry. 55 women were included who presented with preterm labour (regular uterine contractions at least two every 10 minutes associated with cervical change) and intact membranes who required hospitalization before 37 weeks of gestation. This group was divided into women who delivered at term (n=16) (group 1), women without IAI (infection defined as positive amniotic fluid culture for microorganisms and inflammation defined as an amniotic fluid white blood cell (WBC) count >100 cells/mm3) who delivered preterm (n=19) (group 2) and women with preterm labour with IAI who delivered preterm (n=20) (group 3). The second (validation) study included 40, 33 and 40 patients in groups 1, 2 and 3, respectively. Women who delivered preterm without IAI (group 2) had a decrease in both carbohydrates and amino acids whereas women with IAI had AF with less carbohydrates (such as mannose, galactose and fructose) and more amino acids (group 3). A decrease in alanine, glutamine and glutamic acid was noted in patients with preterm labour who delivered at term, while all of these amino acids were increased in the presence of IAI. No explanation was provided for this difference.

In summary, these results implicate that AF PGF2 alpha levels could be of predictive value in the assessment of preterm PROM and the risk for preterm delivery although collection involves an invasive procedure and the studies did not use a control group of children born after 37 weeks. AF PGE2 did not show potential as a biomarker for preterm labour.

### Vagina and Cervix

In total, vaginal and cervical swabs were collected from 1944 women to assess metabolic profiles related to preterm delivery.

#### Nitric Oxide, Nitrate and Nitrite

Nakatsuka et al. (12) collected vaginal secretions from 96 women at 22 to 32 weeks of gestation and investigated the total nitrite and nitrate concentrations. Previous studies from their group showed that the serum concentrations of these two stable metabolites of nitric oxide (NO) are elevated in patients with chorioamnionitis. Four groups were formed: (1) no preterm labour (not further defined) and term delivery (n=54), (2) preterm labour and term delivery (n=16), (3) preterm labour and preterm delivery (n=9) and (4) preterm labour with PPROM and preterm delivery (n=17). Gestational ages at sample collection were 29.2 ± 3.9, 29.7 ± 3.3, 27.0 ± 4.2 and 27.1 ± 3.9 weeks in group 1, 2, 3 and 4, respectively. The total nitrite and nitrate concentrations in the group of women with preterm labour and subsequent preterm delivery was significantly higher than the concentrations in patients who delivered at term, irrespective of preterm labour at the time of sample collection. The total nitrite and nitrate concentrations were also significantly higher in patients with PPROM and preterm delivery.

Vaisanen et al. (13) obtained cervical fluid from 117 women including nonpregnant women (n=11), women seeking termination of pregnancy between 6 and 11 weeks (n=19) and women in late pregnancy (n=87) (with a mean gestational age of 39.7 weeks) in order to sample the cervical fluid NO concentration. Within the late pregnancy group, 37 women were sampled before the start of uterine contractions and 50 women were studied after having been in labour for a mean of 4 hours.

NO was found in the samples of 46% of the nonpregnant women, in 68% of the women in early pregnancy, and in 82% of the women in late pregnancy. The NO-concentration was higher in women in their first trimester compared to nonpregnant women and to term pregnancy with a ripe cervix but without uterine activity than that in term pregnancy with an unripe cervix. Also, the concentration in in parous women was higher than in nulliparous women.

Since NO may be associated with cervical ripening and infectious processes elsewhere in the body (such as periodontal disease) that may play a role in initiating labour, Giannella et al. (14) measured NO levels in plasma, gingival and cervical fluid, which all were sampled simultaneously. NO levels were measured in 820 nulligravid women with low risk of socioeconomic status: 400 cases with preterm labour (defined as uterine contractions>6 contractions in 30 min with documented cervical changes) and 420 controls with normal pregnancy, between 25 + 0 and 33 + 6 weeks of gestation. Women with preterm labour and periodontal disease had the highest gingival and cervical levels of NO, and women with preterm labour showed a worse periodontal status. The highest levels of NO were present in women with preterm labour and subsequent preterm delivery.

##### Lactate and Glucose

Looking at the vaginal fluid of 27 women with PPROM without clinical chorioamnionitis between 22 to 36 (mean= 31.6) weeks of gestation, Dorfeuille et al. (15) measured MMP-8, interleukin-6 and the concentration of the metabolites lactate and glucose. However, the association between glucose and lactate concentration and PPROM was not found after adjustment for gestational age suggesting they are unsuitable metabolites.

##### Ferritin

Ramsey et al. (16) collected cervical fluid and maternal serum from women at 22 weeks (n=2), 23 (n=197) weeks and 24 (n=165) weeks of gestation. This study group focused on the metabolite ferritin. High ferritin levels are associated with acute-phase reactions such as inflammation, and there may thus be an association with elevated serum ferritin concentrations and preterm delivery. Between 22-24 weeks of gestation a serum ferritin value above the 75th percentile was found in 43.5% of women with subsequent spontaneous preterm birth under 32 weeks of gestation versus 10.9% of term controls. Cervical ferritin levels had a weaker association with spontaneous preterm birth under 35 weeks and 37 weeks, suggesting that the correlation is higher in very early preterm delivery especially with serum ferritin.

Broumand et al. (17) analysed ferritin levels in the cervicovaginal fluid using a cervicovaginal swab and the serum levels of ferritin in 280 women between 22 and 24 weeks of gestation and classified subsequent deliveries prospectively into preterm deliveries (<37+0 weeks of gestation), early preterm deliveries (<34+0 weeks of gestation) and very early preterm deliveries (<32+0 weeks of gestation). The mean serum ferritin level was significantly lower in term deliveries versus early and very early preterm deliveries. The ferritin level in cervicovaginal secretions in the term delivery group was significantly lower compared with those with early preterm delivery before 34 weeks of gestational age, however before 32 weeks of gestational age this difference was no longer significant.

##### Metabolic Profiling (Acetate, Lactate, Glucose, Alanine, Succinate and Glutamate/Glutamine) and Microbiome

Stafford et al. (18) combined metabolic profiling with analysis of vaginal microbiota prospectively in women who delivered prematurely compared to term controls in a cohort of asymptomatic women studied at 20–22 weeks of gestation (*n *= 80), 26–28 weeks of gestation (*n* = 41) and symptomatic women (studied at 24–36 weeks, *n* = 37). Symptomatic women presented with imminent but not established, preterm labor (regular uterine contractions but cervix not dilated beyond 3 cm). Mass spectrometry analyses were performed for the metabolites lactate, glucose, acetate, alanine, succinate and glutamate/glutamine. Community composition (community state type, CST) of vaginal microbiota was different in women with term and preterm deliveries. 25% of women delivering preterm had vaginal microflora dominated by *L. jensenii* compared with 10% of the women that delivered at term, suggesting that preterm birth may be associated with *L. jensenii* dominance.

In contrast, two other *Lactobacilli*, L*. crispatus* and *L. gasseri*, might be associated with pregnancies that progress to term. Women who delivered at term had a predominance of *L. crispatus* and *L.gasseri* compared with women who delivered preterm. Elevated vaginal lactate and succinate levels were associated with the predominance of *L. crispatus* and *L.gasseri*. At 26 to 28 weeks of gestation, women who ultimately delivered preterm had significantly lower succinate levels compared to the term women. In addition, significantly higher lactate levels were measured in in samples dominated by *L.crispatus* compared with those dominated by *L.jensenii*.

At 26–28 weeks of gestation, women who ultimately delivered preterm had significantly lower (about 2-fold) succinate levels compared to the term women. This research suggests that *L. jensenii-*dominance could be associated with a higher risk of premature delivery. Also, a decreased level of lactate and/or succinate or the decrease in *Lactobacilli* associated with the presence of these metabolites could be related to the risk of preterm delivery.

##### Acetate

Amabebe et al. (19) evaluated acetate levels with magnetic resonance spectroscopy and enzyme-based spectrophotometry from the cervicovaginal fluid of 82 pregnant women with intact fetal membranes between 24 and 36 weeks of gestation with imminent preterm delivery (defined as regular uterine contractions at least once every 10 min and cervical dilatation <3 cm). 15 of the 82 women delivered preterm (<37 weeks of gestation) and 8 women delivered before 32 weeks of gestation. Acetate was significantly higher in the women who delivered preterm compared with their term counterparts. In addition, elevated cervicovaginal acetate levels were predictive of a delivery within 2 weeks of presentation. However, the predictive effect was not significant under 32 weeks of gestation since there were only 8 women in this subset.

In summary, in cervical and vaginal samples, nitric oxide, nitrate, nitrite, ferritin and acetate have been described to differ in preterm birth and therefore seem have the potential to serve as predictive biomarker for preterm delivery. However, data on these biomarkers have to first be externally validated and sampling conditions need to be standardized before firm conclusions can be drawn on possible implementation in clinical practice. The predictive value of isolated glucose and lactate seems limited, however, the combination of data including microbiome, here different types of lactobacilli, and lactate (and succinate) in the work-up of preterm labour could be promising and need to be assessed in future studies.

#### Blood

Relatively few articles describe metabolites that can be identified in the blood of pregnant women in order to assess preterm delivery. In this review, the results from 3 articles including 211 women are presented.

##### Vitamins A,C,E and 8-Isoprostane

Low ascorbic acid concentrations in blood have been linked to an increased risk of PPROM (20) and the concentrations of cellular membrane 8-isoprostane may serve as a marker of *in vivo* oxidative stress intensity. Ilhan et al. (21) studied the plasma concentration of interleukin-6, C-reactive protein and vitamins A, C, E and 8-isoprostane using ELISA (Enzyme-Linked Immunosorbent Assay) in a total of 72 pregnant women (1) with PPROM and a mean gestational age of 29.2 weeks (n=38) and (2) without PPROM and a mean gestational age of 30 weeks (n=34). In this study, a significant association was found between low 8-isoprostane, low vitamin C and high total oxidant status and the occurrence of PPROM. However, no follow-up was reported as to whether these women also delivered prematurely.

##### (25-Hydroxy)Vitamin D

Akkar et al. (22) measured maternal serum levels of 25-hydroxyvitamin D in pregnant women who presented with preterm labour (between AD 22-37) that resulted in preterm birth (n=35) and preterm labour that resulted in term birth (AD 37-42) (n=44). Preterm labour was defined by uterine contractions (4 every 20 min or 8 every 60 min) with documented cervical change (cervical effacement ≥80% or cervical dilation >2 cm). 25-hydroxyvitamin D presents a storage form of vitamin D, and an association between vitamin D status and adverse pregnancy outcomes such as preterm birth, has earlier been suggested. Vitamin D is active in the immune response (involving activation of natural killer cells and monocytes), decreases inflammation and prevents bacterial infections. Pregnancies deficient of vitamin D show an increased production of tumour necrosis factor-α-like inflammatory cytokines and thus an increased immune response. In this study, there was a decreased level of serum 25-hydroxyvitamin D levels in the preterm birth group. In contradiction, Bhupornvivat et al. (23) did not see a difference in the serum 25-OHD concentrations (when matched for gestational age), vitamin D deficiency and insufficiency between a preterm labour (n=30, with a gestational age between 24 + 0 – 36 + 6 weeks) and the term labour control group (n=30).

Summarizing, the occurrence of PPROM is probably related to a high total oxidant status and low vitamin C, however there is no evidence that this leads to preterm labour. Vitamin D shows conflicting results as a predictive metabolite.

#### Urine

The urine of 496 pregnant women was investigated in two different articles included in this review for metabolites predicting preterm labour.

##### Urinary Phthalate and Serum 8-Isoprostane

Ferguson et al. (24) investigated the connection between urinary phthalate metabolites and biomarkers of oxidative stress such as 8-isoprostane. Phthalates, found in a variety of products such as enteric coatings of pharmaceutical pills, gelling agents, stabilizers and emulsifying agents to name a few, have been linked to adverse birth outcomes such as preterm delivery.

For the metabolite 8-isoprostane, 58 patients were sampled for both urine and blood three times during pregnancy, at 16-20 weeks of gestation, 20-24 weeks of gestation and at 24-28 weeks of gestation. The phthalate metabolites were associated with increases in biomarkers such as 8-isoprostane, which are associated with oxidative stress. This in turn could have an association with preterm delivery. However, the study did not report delivery dates and did not include knowledge on intrauterine infection.

##### Lysine and Formate

Maitre et al. (25) used a more extensive metabolic profiling approach by sampling the metabolites lysine and formate from urine of pregnant women (n=438) at the end of the first trimester (mean gestational age 11.96 weeks) and following the women up until delivery. This study looked at negative birth outcomes such as preterm birth (defined as before 37 + 0 weeks of gestation) but focused mainly on fetal growth restriction. 114 women delivered preterm (mean gestational age 35 + 5 weeks). Significantly elevated urinary lysine and decreased formate levels determined during the first trimester were found in women who spontaneous delivered preterm at a later stage in the same pregnancy. Lysine is also elevated in the plasma of premature infants.

In conclusion, one can only assume a biomarker of oxidative stress such as 8-isoprostane could lead to premature delivery since Ferguson et al. (24) does not report this outcome. Urinary lysine and formate measured at the end of the first trimester could in contrast be used to predict negative birth outcomes.

#### Placenta & Chorionic and Amniotic Membranes

Metabolites found from the placenta cannot serve as predictive biomarker for preterm labour, because they can only be obtained after birth, unless they can also be detected in maternal blood. This review describes the outcome of six studies, including 176 placentas that were all collected postpartum when the outcome of delivery was known.

##### Prostaglandin E2

Furuta et al. (26) obtained placentas from women undergoing primary caesarean sections at full term, separated the amnion of the chorion, and isolated the amnion cells. The amniotic membranes were obtained from 9 women prior to the onset of labour or rupture of the fetal membranes. The PGE2 production in culture supernatant was measured using an enzyme immunoassay method. Five inflammatory cytokines, i.e. interleukin (IL)-1, IL-1ß, IL-6, IL-8 and tumour necrosis factor-(TNF-alpha) stimulated PGE2 production from amnion cells. This suggests that for example following IAI, inflammatory cytokines directly stimulate PGE2 production from amnion cells and may initiate premature labour (or rupture of membranes). However, PGE2 production was not measured in maternal serum and this study was conducted in full term pregnancies.

##### Progesterone

Garcia-Ruiz (27) obtained 10 placentas under similar conditions from women during caesarean section. After pre-treatment with different concentrations of progesterone (1.0 μM, 0.1 μM, and 0.01 μM) of progesterone for 24 h, then fresh medium was added including co-stimulations with 1000 ng/ml of LPS plus 0.01, 0.1, and 1 μM of progesterone) the placentas were stimulated with lipopolysaccharide (LPS) of *Escherichia coli*. LPS stimulation caused a significant increase in the level of all cytokines, however, pre-treatment with progesterone suppressed the secretion of TNF-alpha and interleukins. With the highest concentration of progesterone, MMP-9 was inhibited. Progesterone could therefore be part of the compensatory mechanism that limits the cytotoxic effects associated with an infection. Measuring progesterone levels could be interesting in combination with other biomarkers to evaluate their protective effect on infection and hypothetically, preterm delivery.

##### Adenosine Monophosphate-Activated Kinase

Lim et al. (28) studied adenosine monophosphate (AMP)-activated kinase (AMPK), which is involved in the prevention of inflammation. Amongst others, pro-inflammatory cytokines and MMP-9 play a central role in the rupture of fetal membranes and a possible inhibition initiated by AMPK in this process has not yet been studied. Thus, this study group obtained fetal membranes from non-labouring women at term undergoing elective caesarean section (n=6) and term women after spontaneous labour/spontaneous membrane rupture/normal vaginal delivery (n= 6). The group also obtained fetal membranes from preterm women without histological chorioamnionitis from three groups: (1) non-labouring undergoing caesarean section with intact membranes (n=6), (2) non-labouring undergoing caesarean section with PROM (n=6) and (3) after spontaneous labour and normal vaginal delivery (n=6). Amnion cells stimulated with interleukin-1b were used to investigate the effect of AMPK activators on MMP-9 expression and secretion. AMPK activity was decreased with term labour and in PROM compared to intact membranes. This could suggest that AMPK plays a role in the rupture of membranes however without onset of labour.

##### 4-Phenylbutyric Acid and Tauroursodeoxycholic Acid

Liong et al. (29) obtained fetal membranes from term non-labouring women undergoing caesarean sections (n=8), term women after spontaneous labour/spontaneous membrane rupture and normal vaginal delivery (n=8) and preterm non-labouring women undergoing caesarean section (n=10), after spontaneous labour and normal vaginal delivery (n=10) and after spontaneous labour and normal vaginal delivery with histologically confirmed chorioamnionitis (n=8).The aim was to determine the effect of the endoplasmic reticulum (ER) stress inhibitors 4-phenylbutyric acid (4-PBA) and tauroursodeoxycholic acid (TUDCA) on LPS-induced prolabour mediators in fetal membranes and myometrium. Tissues were pretreated with 4-PBA or TUDCA before the addition of LPS derived from *Escherichia coli*. ER stress of the placenta has been described in women with intrauterine growth restriction but the role of ER stress in human labour has not been established. The study showed that the use of 4-PBA and TUDCA alleviated ER stress induced by LPS and ameliorated the increase in LPS-induced prolabour mediators.

##### NAD(P)H Oxidase

Matsubara et al. (30) studied the chorioamniotic membranes of 15 Japanese women, five of whom delivered between 32-38 weeks of gestation, without signs of chorioamnionitis on routine istological examination and five of whom delivered between 30-39 weeks of gestation with signs of infection (such as elevated C-reactive protein, leucocytosis and a subfebrile temperature up to 37.5 degrees Celsius), had regular uterine contractions of at least 10 min intervals and had a histological diagnosis of chorioamnionitis, and five pregnant women who delivered between 28-37 weeks of gestation who had severe chorioamnionitis by histological examination and clinical signs of infection (elevated C-reactive protein and leucocytosis). Subcellular localizations of NAD(P)H oxidase, a reactive oxygen species producing enzyme, was examined using ultrastructural enzyme histochemistry. The presence of NAD(P)H oxidase is strong evidence of phagocytic cell activation, which may play a role in the defence of chorioamniotic membranes against infection and in the pathogenesis of chorioamnionitis-related preterm delivery.

Indeed, fetal membranes without chorioamnionitis showed 25% NADPH oxidase activity and in mild chorioamnionitis, fetal membranes showed 51% NADPH oxidase activity.

##### Ascorbic Acid and Collagen

Stuart et al. (20) studied the fetal membranes of 37 women with term PROM and another group of 37 women whose membranes had ruptured spontaneously during term labour in order to see whether ascorbic acid status modulates reduced collagen concentrations in PROM. Ascorbic acid is vital for the production, modulation and maintenance of collagen and also has an important antioxidant function. The fluorometric assay of the fetal membranes of women with PROM showed a significantly lower concentration of ascorbic acid compared with the control women. However, there was no difference in ascorbic acid concentrations in the maternal circulation between PROM and control women. Also, the concentration of collagen in amniotic membrane showed no significant association with amniotic-membrane ascorbate concentration.

In summary, in placenta and membranes the metabolites progesterone, AMPK, 4-PBA, TUDCA, NADPH-oxidase, ascorbic acid and collagen have been studied to retrospectively evaluate whether there was a connection with chorioamnionitis/PROM. None of these metabolites can be used in the prediction of preterm labour since they were only collected from the placenta and/or membranes.

#### Umbilical Cord Blood

Similarly, umbilical cord blood can only easily be collected postpartum (unless a chordocentesis is performed) and therefore cannot be used as a non-invasive marker in the prediction of preterm labour. We include one study that also describes an ultrasound evaluation in combination with the metabolite cortisol in the assessment of preterm labour.

##### Cortisol and Dehydroepiandrosterone

Finally, Buhimschi et al. (31) conducted a prospective study in 51 consecutive fetuses of mothers who had a clinically indicated amniocentesis (to rule out infection or inflammation) and looked at the correlation of fetal adrenal gland size and levels of the metabolites cortisol and dehydroepiandrosterone (DHEAS) in umbilical cord blood postpartum. All women included in this study presented with advanced cervical dilatation (more than 3 cm), preterm labour (defined as regular uterine contractions associated with advanced cervical dilatation or effacement) or preterm premature rupture of the membranes (PROM). Eligible women were pregnant of a singleton fetus at less than 34 + 0 weeks of gestation. Several studies have reported an increase of cortisol at the initiation of delivery. It is recognized that levels of maternal cortisol increase especially in vaginal deliveries compared with elective caesarean section, however, controversy exists on the role of cortisol in preterm labour or intrauterine infection (32). Buhimschi et al. (31) found that fetal adrenal gland volume neither reflected the fetal circulating levels of cortisol or DHEAS. The median umbilical cord concentrations of cortisol and DHEAS were not significantly different between women who had an intraamniotic infection (n=16) and women without (n=35). However, cord blood levels in this study were measured retrospectively and not *via* antenatal chordocentesis, which in turn could also affect cortisol levels.

## Discussion

### Summary of Main Findings

In premature delivery, biomarkers such as cytokines and chemokines have been analysed in diverse biological fluids (amniotic fluid, urine, cervical/vaginal fluid, serum and plasma) to understand associations with the mechanism of onset of labour. Many of these markers, especially if related to inflammation or infection are found in women with preterm labour however few have shown high either positive or negative predictive potential in clinical practice (1). Biomarkers such as matrix metalloproteinase-9 and oestriol have shown an association with a risk of preterm delivery, however they are late predictors and are of little use in timely prediction for prevention in clinical practice. Thus far, the best biochemical marker for preterm birth is fetal fibronectin, which represents choriodecidual disruption and has a high negative predictive value. A Cochrane review from 2019 (33) concurs that fibronectin may be used to predict and therefore reduce deliveries before 37 weeks, however cost effectiveness analyses are needed and the evidence was found to be of low quality. More accurate biomarkers are urgently needed.

The results presented in the current systematic review are conflicting. Vaginal or cervical swabs could be easy to implement into the clinical setting to predict imminent preterm labour and the results are somewhat promising. Nakatsuka et al. (12) showed that total nitrite and nitrate concentrations in the group of women with preterm labour (sampled between 22 and 32 weeks) and subsequent premature delivery was significantly higher than the concentrations in patients who delivered at term. Vaisanen et al. (13) showed that cervical fluid nitric oxide in late pregnancies with a ripe cervix without uterine activity was higher than that in a term pregnancy with an unripe cervix. However, there are confounders that both studies did not correct for, such as the presence of nitric oxide in semen. The mean value of the seminal NO concentration and the time-lapse since the latest sexual intercourse are variables that should be accounted for during cervical fluid sampling. Further cut-off values for cervical samples would also be needed for parous vs nulliparous women since baseline values were higher in multiparous women with a riper cervix to begin with. Parity was not a factor in plasma NO levels.

However, contradictory data has been published for NO since its role as a prognostic test contradicts with the way nitroglycerin is used in practice, namely to relax the uterus. A Cochrane review from 2014 investigated the role of routine administration of NO as a treatment for imminent preterm birth (34). Animal experiments have shown that the inhibition of NO increases myometrial contractility and NO synthase activity is increased during pregnancy and reduced at the onset of labour (34). In the review, NO in the form of intravenous/transdermal or sublingual drugs were given in 12 randomised controlled trials (n=1227). NO did not delay delivery more than 48 hours in comparison to other tocolytics (such as for example betamimetics and calcium channel blockers) and is not recommended for routine administration. However, the role of NO in the developing pregnancy and parturition clearly needs to be studied further.

The role of ferritin in predicting preterm birth was also assessed in this systematic review. Ferritin is involved in inflammatory pathology through its role in the innate immune response and is currently used as a biomarker of disease progress and prognosis (for example in rheumatology and hematology) as well as a target for therapeutic interventions (35). The precise mechanism by which ferritin contributes to disease is unclear, though. Ramsey et al. (16) saw a significant association between elevated cervical ferritin levels and subsequent spontaneous preterm birth along with Broumand et al. (17), who also investigated serum levels of ferritin. However, cervicovaginal ferritin correlated poorly with serum ferritin levels as well as serum inflammatory markers such as interleukin 6. Also, serum hyperferritinemia is a very specific condition since it can be elevated due to inflammation elsewhere in the body, not just due to imminent labour. Cervical ferritin could however potentially be used in combination with other markers for the prediction of preterm delivery.

One of such markers could be acetate, which was elevated in the cervicovaginal fluid of women delivering between 32 and 36 weeks in the study conducted by Amabebe et al. (19). Maitre et al. (25) obtained urine samples at the end of the first trimester and saw significantly elevated levels of lysine and decreased levels of formate in women with spontaneous preterm delivery, however no significant difference was seen between acetate levels. Obviously, the sample were of a different origin in the two trials, and Maitre used a very specific cohort, so the results would have to be validated elsewhere, too.

However, the background of these trials provides further food for thought. Acetate is produced by anaerobic bacteria of the female genital tract and increases the vaginal pH. This in turn encourages the growth of potentially harmful bacteria and induces inflammation and infection. In contrast, an increase in lactate (produced by most of the *Lactobacillus* species) leads to a decrease in vaginal pH.

Future research should include a combination of metabolic profiles with analysis of the local set of vaginal microbiota (18). This approach should include a detailed analysis of associations between certain metabolites and the presence of specific bacterial species, since the abundance of a certain species may not necessarily correlate with the amount of metabolite detected (36).

Akkar et al. (22) observed a significant decrease in maternal serum 25-hydroxytamin D levels in spontaneous preterm birth, however in the study by Bhupronvivat et al. (23) these levels were not different between the preterm and control group. Vitamin D is already recommended as a supplement by the WHO during pregnancy because it may reduce the risk of pre-eclampsia, low birthweight and preterm delivery (37). This advice is based on a Cochrane review from 2016 where, for preterm birth, patients from three trials (n=477) were included and showed a reduced risk for preterm birth with vitamin D supplementation compared to no intervention or placebo (8.9% vs 15.5% RR 0.36, CI 0.14-0.93) (38). However, the level of this evidence is of moderate quality. Different studies used different doses of 25-hydroxyvitamin D supplementation and different methods to assess serum 25-hydroxyvitamin D (the best method being high performance liquid chromatography mass spectrometry but only one trial used this method in the whole review). More research is clearly needed to advance the interpretation of these findings and integration into clinical practice.

Could prostaglandin levels measured in AF predict preterm delivery? Looking at the study conducted by Lee et al. (8), prostaglandin could have prognostic value in patients presenting with preterm PPROM, however it is possible that there is another mechanism for the increase in prostaglandin, such as the activation of the hypothalamic pituitary adrenal (HPA) axis of the fetus. Studies in animals have shown that the activation of the HPA-axis leads to an increase in cortisol, leading to an altered ratio of progesterone:estrogen and thus an increase in prostaglandin levels. In the last 10-15 days before delivery, glucocorticoids induce maturation of fetal organs such as the lungs and liver. In ovine parturition, there is a progressive increase in plasma, amniotic fluid and intrauterine tissue concentration of prostaglandin, especially PGE2 after activation of the HPA-axis (39). However, Buhimschi et al. (31) did not see an evident increase in cortisol levels of fetuses born at less than 34 weeks of gestation. Interestingly though, not all studies in humans show this increase in prostaglandin in premature labour, for example Romero et al. (11) did not see a prostaglandin level difference in his metabolic profiling study of AF albeit not defining the type of prostaglandin investigated.

### Limitations

The different research groups investigated a diversity of populations with various control groups, used varying methods to identify metabolites (e.g., immunoassay, mass spectrometry) and different approaches were practiced to obtain AF(where some relied on amniocentesis and others the collection of amniotic fluid after ruptured membranes), making comparison between these extremely difficult. In addition, it is already known that ethnic and sex differences in metabolite profiles exist, with different metabolic associations (40–42). Furthermore, an amniocentesis is not a riskless procedure, subjective to the examiner and the question is whether this should be used in the diagnostic work-up for the prediction of preterm labour if the predictive potential has yet to be proven. Since there is a need for a fast, non-invasive biomarker, AF as a predictive source of metabolites to detect preterm birth should be disregarded.

Studies involving fetal membranes are challenging to translate into the clinical setting. Placentas from mostly primary caesarean sections were investigated and thus cannot be used to predict premature labour. The research conducted by Lim et al. (28) could be translated to the clinical setting, if AMPK activators would be studied as a possible therapeutic for preterm labour. Also, further research needs to be conducted into role of 4-PBA and TUDCA in labour. On histological examination, NADPH oxidase levels are increased in chorioamnionitis-related preterm delivery, however this is also a postpartum finding. A recent systematic review suggests that there is an overall increase in oxidative stress in relation to preterm birth (43). The role of vitamin C as a suppressor of oxidative stress at delivery and in the production of collagen in fetal membranes has been suggested. Stuart et al. proposes vitamin C plays a role in the breaking of membranes, however this finding does not translate to serum samples (20).

Although of great interest for future research, we did not include maternal fecal metabolites or the fecal microbiome in the clinical workup of preterm birth in the original search as this was beyond the scope of our review. However, Gough et al. (44) recently demonstrated that the presence of *Slackia isoflavonivonvertens* in fecal samples of 207 pregnant women (22.8% of women in their first trimester, 60.9% in their second trimester and 16.3% in their third trimester) could contribute to a longer gestational age. Importantly, the presence of *Prevotella copri*, a microbe associated with host inflammation, was prospectively detected more frequently in women who delivered preterm. In a case-control study by Gershuni et al. (45) matching samples for race and maternal obesity, spontaneous preterm delivery was associated with both the presence of *Betaproteobacteria* and lipid profiles in fecal samples. The consumption of saturated fat and the presence of docosahexaenoic acid and eicosapentaenoic acid in fecal samples correlated with spontaneous preterm delivery. The role of diet and the metabolites associated with different bacteria could thus potentially be used as a predictive tool in pregnant women in the future if these results are validated in a larger group.

### Future Perspectives and Conclusion

One focus of future research could be a non-invasive method using volatile organic compounds (VOCs) to detect imminent premature labour based upon mixtures of preterm birth-associated as well as physiological VOCs, as touched upon briefly in the introduction of this review.

An electronic, mobile, handheld device (e-nose) could be used to detect VOCs reflecting alterations of a local and systemic metabolic state, as caused by subclinical infection, inflammation or alterations of the local microbiota.

Lacey et al. (46) showed that VOC analysis of vaginal swabs obtained in the mid trimester, has potential in the prediction of preterm delivery, with a sensitivity of 0.66 (95%CI 0.56–0.75) and specificity 0.89 (95%CI 0.82–0.94). However, in order to validate the e-nose as a diagnostic tool, studies using elaborate techniques such as liquid chromatography and mass spectrometry, allowing for detection of individual VOC molecules rather than the pattern-recognition based are needed. Identification of a set of specific VOCs associated with preterm birth may allow for development of tailor-made e-nose sensors aimed at recognition of these VOCs. Advantages of e-nose technology include low costs and bed-side application of non-invasive analysis of vaginal swab or urine sample (as opposed to for example blood or amniotic fluid). Also, the biomarkers with most potential, such as nitrate, ferritin and acetate should be validated in an external cohort.

In conclusion, there is an increasing focus on research into non-invasive biomarkers of premature birth, however without universal consensus. Future research should focus on external validation of potential biomarkers in vaginal swabs and urine of pregnant women and look at a combination of the most promising ones.

## Data Availability Statement

Publicly available datasets were analyzed in this study. The references include all articles used for this review.

## Author Contributions

ER, IR, and SS contributed to the design and implementation of the research. IR, TH, TM, NF, and SS contributed to the analysis of the results. ER wrote the manuscript and all authors discussed the results and commented on the manuscript. All authors contributed to the article and approved the submitted version.

## Funding

This research was funded by the department of Obstetrics and Gynecology of the Erasmus Medical Center, Rotterdam.

## Conflict of Interest

The authors declare that the research was conducted in the absence of any commercial or financial relationships that could be construed as a potential conflict of interest.

## Publisher’s Note

All claims expressed in this article are solely those of the authors and do not necessarily represent those of their affiliated organizations, or those of the publisher, the editors and the reviewers. Any product that may be evaluated in this article, or claim that may be made by its manufacturer, is not guaranteed or endorsed by the publisher.
